# A QS21 + CpG-Adjuvanted Trivalent HSV-2 Vaccine and Trivalent HSV-2 mRNA Vaccine Induce a Strong Immune Response, Protect Against HSV-2 Infection, and Cross-Protect Against HSV-1 Infection in Mice

**DOI:** 10.3390/vaccines13050497

**Published:** 2025-05-06

**Authors:** Han Cao, Xiaolong Zhang, Jishuai Cheng, Yang Li, Ning Luan, Jingping Hu, Bingyan Liang, Haihao Zhang, Dandan Gao, Zhentao Lei, Yufeng Yao, Cunbao Liu

**Affiliations:** 1 Institute of Medical Biology, Peking Union Medical College, Chinese Academy of Medical Sciences, Kunming 650118, China; caohan@imbcams.com.cn (H.C.); zhangxiaolong@imbcams.com.cn (X.Z.); luanning@imbcams.com.cn (N.L.); hujingping@student.pumc.edu.cn (J.H.); s2023018029@student.pumc.edu.cn (B.L.); zhanghh@imbcams.com.cn (H.Z.); ddgao2008@imbcams.com.cn (D.G.); s2023018021@student.pumc.edu.cn (Z.L.); 2 Laboratory Animal Department, Kunming Medical University, Kunming 650500, China; chengjishuai@kmmu.edu.cn (J.C.); liyang3@kmmu.edu.cn (Y.L.)

**Keywords:** herpes simplex virus, trivalent antigen, subunit vaccine, mRNA vaccine, cross-protection

## Abstract

Background: HSV-2 infection continues to be a significant global health concern, as there are no approved vaccines despite numerous attempts at development. Methods: This study explored the immunogenicity and protective efficacy of aluminum- or QS21 + CpG-adjuvanted trivalent HSV-2 vaccines and a trivalent HSV-2 mRNA vaccine incorporating the gC2, gD2, and gE2 antigens. Results: Our results demonstrated that the QS21 + CpG-adjuvanted subunit vaccine and mRNA vaccines successfully induced robust antigen-specific humoral and cellular immune responses and provided significant protection against both HSV-2 and HSV-1 infection. These vaccines showed remarkable efficiency in reducing the viral load and preventing clinical symptoms in mice, highlighting their potential for clinical application. Conversely, the aluminum-adjuvanted vaccine exhibited limited effectiveness, emphasizing the superiority of the QS21 + CpG-adjuvanted and mRNA vaccines. Conclusions: These findings provide valuable insights for the continued development of effective HSV vaccines and suggest promising strategies for preventing both HSV-2 and HSV-1 infection.

## 1. Introduction

Herpes simplex virus (HSV), a prevalent pathogen in the alpha subfamily of the Herpesviridae family, is divided into two serotypes, HSV-1 and HSV-2, on the basis of antigenic differences [1]. HSV-1 infection leads to oral herpes, genital herpes, herpetic keratoconjunctivitis [2], and encephalitis [3]. According to the World Health Organization (WHO), approximately 3.8 billion people under 50 years of age (64.2%) are infected with HSV-1 globally [4]. HSV-2 infections are a common cause of genital herpes [5]; it can also lead to neurological complications such as meningitis and encephalitis [2]. Alarmingly, HSV-2 infection is closely associated with an increased incidence of HIV [6,7], which poses greater health risks to individuals. There were approximately 19.2 million new HSV-2 infections worldwide, affecting 0.5% of the global population [4], highlighting an urgent need for an effective vaccine.

The vaccines developed to date mainly target glycoprotein D (gD), which is crucial for recognizing host receptors, facilitating viral adsorption, and triggering membrane fusion [8]. However, clinical trials have shown that single-component gD vaccines offer limited protection [9,10]. Therefore, in this study, we included two more glycoproteins, gE and gC, as extra components of the HSV-2 vaccine. gE forms heterodimers with gI, mediates the directional transport of virions to epithelial cell junctions, and thus promotes cell-to-cell spread [11]. Moreover, gE can bind the Fc segment of host IgG, inhibiting antibody-dependent cellular cytotoxicity (ADCC) mediated by Fcγ receptors [12,13] and helping the virus evade host immune surveillance. gC binds complement component C3b, blocking the classical and alternative complement pathways, preventing membrane-attack complex formation, protecting infected cells from lysis, and impairing B- and T-cell immune functions [14,15,16]. The combined use of gD, gE, and gC glycoproteins as antigens will diversify the immune response induced by the vaccine, providing the body with more comprehensive protection against invading viruses.

Subunit vaccines often lack pathogen-associated molecular patterns (PAMPs) to activate pathogen recognition receptors (PRRs), resulting in insufficient innate immune activation and subsequently weak adaptive immune response. Thus, adjuvants bridging innate and adaptive immunity are needed to increase immunogenicity [17]. CpG oligodeoxynucleotides (ODNs), which contain unmethylated CpG motifs, are recognized by TLR9 and activate innate immune response [18,19]. QS21, a saponin adjuvant from *Quillaja saponaria*, has strong immune-stimulating activity [20]. CpG and QS21 are components of approved vaccines such as Heplisav-B [21] and Shingrix [22]. In research on VZV vaccines, both humoral and cellular immune responses were enhanced when CpG and QS21 were used in combination as adjuvants compared with when either was used alone [23]. HSV establishes latent infections with periodic reactivation, posing significant challenges for antibody-mediated clearance. An ideal HSV vaccine should effectively activate cell-mediated immunity (CMI) to eliminate virus-infected cells, thereby curbing viral replication and spread. QS21 and CpG, which are known to induce a robust cellular immune response, are particularly advantageous in this context. These adjuvants activate both the innate and adaptive immune systems, generating elevated levels of antigen-specific T cells. The synergistic effect of these adjuvants likely arises from their capacity to stimulate multiple innate immune pathways, including the Toll-like receptor (TLR) and NOD-like receptor (NLR) families, resulting in a more comprehensive adaptive immune response. While the role of CpG has been extensively studied in HSV vaccine research [24,25,26], the combination of CpG and QS21 as adjuvants in HSV-2 subunit vaccines remains underexplored. Our study underscores the potential of this adjuvant combination to enhance vaccine efficacy against both HSV-2 and HSV-1 infections.

This study aimed to evaluate the immunogenicity of trivalent HSV-2 vaccines targeting the antigens gE, gD, and gC, in the forms of QS21 + CpG-adjuvanted subunit vaccine, mRNA vaccine, or traditional aluminum-adjuvanted vaccine. This study also determined their effectiveness in preventing HSV-2 infection and providing cross-protection against HSV-1 infection. Our results indicated that both the QS21 + CpG-adjuvanted subunit vaccine and mRNA vaccine induced strong humoral and cellular immune responses. The QS21 + CpG-adjuvanted subunit vaccine outperformed the mRNA vaccine in inducing gE-specific antibodies, while the aluminum-adjuvanted subunit vaccine showed poor immunogenicity, failing to elicit sufficient antibody and cell mediated immunity (CMI) responses. The QS21 + CpG-adjuvanted subunit vaccine and mRNA vaccine, but not the aluminum-adjuvanted subunit vaccine, provided effective protection against lethal HSV-2 and HSV-1 challenges.

## 2. Materials and Methods

### 2.1. Vaccine Preparation

In this study, two HSV-2 subunit vaccines and one HSV-2 mRNA vaccine were prepared. The vaccine groups and doses per injection are shown in Table 1. The antigen of the subunit vaccine consisted of the HSV-2 gE, gD, and gC proteins (AtaGenix Laboratory Co., Ltd., Wuhan, China) in equal mass ratios (2.5 μg each); Chinese hamster ovary (CHO) cells were used to express the proteins, and a nickel column was used to purify the proteins. Before injection, the subunit vaccines were mixed with either an aluminum hydroxide (Thermo Fisher, Carlsbad, CA, USA) or a combination of CPG 1018S (5′-TGACTGTGAACGTTCGAGATGA-3′) (Sangon Biotech, Shanghai, China) and QS21 (Alpha Diagnostic Intl. Inc., San Antonio, TX, USA).

The mRNA vaccine was formulated by encapsulating mRNA encoding the HSV-2 gE, gD, and gC proteins in equal proportions (5 μg each) within lipid nanoparticles (LNPs). The DNA sequences encoding full-length HSV-2 gE, gD, and gC were codon-optimized and synthesized. After adding 5′ UTR, 3′ UTR, and polyA sequences, these sequences were cloned and inserted into the pBlueScript II SK(+) plasmid (AtaGenix Laboratory Co., Ltd., Wuhan, China). Linearized DNA templates were used for in vitro transcription with a T7 High Yield RNA Transcription Kit (N1-Me-Pseudo UTP) (Vazyme Biotech Co., Ltd., Nanjing, China) to produce mRNA, which was then purified using VAHTS RNA Clean Beads (Nanjing Vazyme Biotech Co., Ltd.) to remove contaminants. The mRNA concentration was quantified using a Quant-iT™ RiboGreen^®^ RNA Reagent and Kit (Thermo Fisher, Carlsbad, CA, USA), and mRNA-LNP complexes were prepared as previously described [27]. Briefly a three-component mRNA mixture was prepared by combining HSV-2 gE, gD, and gC mRNA in a 1:1:1 ratio in 100 mM citric acid-citrate solution (pH 4.0). The LNP lipid phase (Sinopeg Biotech Co., Ltd., Xiamen, China) was prepared by dissolving ionizable cationic lipids, DSPC, cholesterol, and PEGylated phospholipids in anhydrous ethanol at a molar ratio of 46.3:9.4:42.7:1.6. Using a microfluidic device (Precision Nanosystems, Vancouver, BC, Canada), the water and lipid phases were mixed at a 3:1 ratio. The mixture was then ultrafiltrated with DEPC-treated PBS and DEPC-treated PBS containing 8% sucrose to remove excess ethanol and concentrate the product. The final mRNA-LNP complexes were filtered through a 0.22 μm syringe filter (Millipore, Burlington, MA, USA) and stored at −80 °C. The particle size and polydispersity index (PDI) of the mRNA-LNP complexes was measured with a particle size analyzer (Malvern, Worcestershire, UK). The mRNA concentration in the water phase before and after encapsulation was determined using the Quant-iT™ RiboGreen^®^ RNA Reagent and Kit (Thermo Fisher, Carlsbad, CA, USA) after overnight lysis in 1% Triton at 4 °C. The encapsulation efficiency was calculated accordingly. The integrity of the mRNA samples was assessed via 1% formaldehyde denaturing agarose gel electrophoresis.

### 2.2. Animal Studies

Female BALB/c mice (3–4 weeks old and specific pathogen-free (SPF)-grade) were purchased from Vital River Laboratory Animal Technology Ltd. (Chengdu, China) and housed at the Animal Experiment Department of IMBCAMS in a barrier environment. Mice were immunized intramuscularly twice at 3-week intervals (50 μL per injection). One week after secondary immunization, blood was collected, the serum was isolated, the antibody titers were determined, neutralizing antibodies were detected, and the spleens were harvested for cellular immune assays as previously described [27]. Four weeks after secondary immunization, the mice were challenged with wild-type HSV-2 (HG52) or HSV-1 (Mckrae) virus to assess the protective effect of the vaccines. For the HSV-2 challenge, three weeks following secondary immunization, mice were intramuscularly injected with medroxyprogesterone acetate (3 mg per mouse) to modulate their physiological cycle and increase their susceptibility to HSV-2. Five days post-injection, the mice were vaginally challenged with HSV-2 at a dose of 1.2 × 10^5^ PFU. For the HSV-1 challenge, mice were intranasally inoculated with 2.4 × 10^5^ PFU of HSV-1. The mice were monitored daily post-challenge for weight changes, clinical symptoms, and survival rates. All animal experiments conducted in this study were approved by the Ethics Committee of Animal Care and Welfare of the IMBCAMS (permit number DWSP202410007/DWSP202410016) and complied with the animal ethics guidelines of the Office of Laboratory Animal Management of Yunnan Province, China.

### 2.3. Enzyme-Linked Immunosorbent Assay (ELISA)

The levels of antibodies against HSV-2 gE, gD, and gC in serum were determined via ELISA. Briefly, for each antigen, 96-well microplates (Corning, Corning, NY, USA) were coated with purified protein (2 μg/mL) and incubated overnight at 4 °C. After being blocked with 5% (*w*/*v*) skim milk in PBS for 1 h at 37 °C, the plates were washed with PBS containing 0.05% (*v*/*v*) Tween-20 (PBST). Serum samples were serially diluted and added to the plates, followed by incubation for 2 h at room temperature. The plates were then incubated with a 1:10,000 dilution of horseradish peroxidase (HRP) conjugated goat anti-mouse IgG for 1 h at 37 °C. After washing, 3,3′,5,5′-tetramethylbenzidine (TMB) was added, and the reaction was stopped after 5 min with 2 mol/L sulfuric acid. The absorbance at 450 nm was measured using a spectrophotometer (BioTek Instruments, Inc., Winooski, VT, USA). The IgG titers were defined as the maximum dilution showing an absorbance greater than 0.15. IgG titers that displayed an OD450 lower than 0.15 at a dilution of 1:1000 were considered 100 for calculations.

### 2.4. Neutralization Assays

Mice sera were inactivated at 56 °C for 30 min and then serially diluted in 96-well plates. HSV-2 or HSV-1 virus was diluted in serum-free and antibiotic-free Dulbecco’s modified Eagle’s medium (DMEM) and 100 PFU of the virus was incubated with serum at a 1:1 ratio in a 37 °C, 5% CO_2_ incubator for 2 h. After incubation, the virus–serum mixture was added to a Vero cell suspension diluted to a concentration of 1.5 × 10^5^/mL in DMEM containing 2% newborn bovine serum and then incubated at 37 °C in a 5% CO_2_ incubator for 5–7 days to assess cytopathic effects.

### 2.5. Analysis of Cytokines Secreted into the Supernatant of Splenocytes

For cytokine quantification, splenocytes (1 × 10^6^ cells per well) were stimulated with a mixture of the gE, gD, and gC proteins (15 μg/mL, mixed at a 1:1:1 ratio) or PMA/ionomycin as a positive control for 24 h at 37 °C in 5% CO_2_. Supernatants were collected, and the levels of IL-2 and IFN-γ were measured using ELISA. Standard curves were constructed using the absorbance values and concentrations of the standards to calculate the cytokine concentrations in the samples.

### 2.6. Enzyme-Linked Immunospot Assay (ELISPOT)

ELISpot plates (Merck & Co., Inc., Kenilworth, NJ, USA) were pretreated with 75% ethanol and subsequently washed with PBS. Anti-IL-2 and anti-IFN-γ antibodies (Invitrogen, Waltham, MA, USA) were diluted in PBS and added to the plates at a concentration of 2 μg/mL, followed by overnight incubation at 4 °C. After washing with complete RPMI 1640 (RPMI 1640 with 10% fetal bovine serum and 1% penicillin-streptomycin), fresh complete RPMI 1640 was added to the plates for 2 h at room temperature for blocking. The isolated splenocytes were suspended in serum-free ELISpot medium (Dakewe Biotech Co., Ltd., Shenzhen, China) and then added to the plates at a density of 3 × 10^5^ cells per well. A mixture of gE, gD, and gC proteins (30 μg/mL; 1:1:1 ratio) was added to the experimental wells; PMA/ionomycin (Dakewe) was added to the positive control wells, and blank medium was added to the negative control wells. The 96-well plates were cultured statically overnight at 37 °C and 5% CO_2_; 24 h later, plates were washed with cold deionized water and PBS. Biotinylated anti-mouse IL-2 and anti-mouse IFN-γ antibodies (1 μg/mL; Invitrogen, Waltham, MA, USA) were then added to the plates, which were subsequently incubated for 3 h at room temperature. Following washes with PBS, diluted HRP-conjugated streptavidin (1:1500, Biolegend, San Diego, CA, USA) was added, and the plates were incubated for 1 h at room temperature in the dark. Finally, an ELISpot assay kit (Dakewe) was used to visualize the spots according to the manufacturer’s instructions. Plates were read and photographed using an ELISPOT reader system (Autoimmune Diagnostika GmbH, Strassberg, Germany).

### 2.7. Flow Cytometry Analysis

The reagents and antibodies used for flow cytometry were obtained from BioLegend (San Diego, CA, USA). Splenocytes were plated in 24-well plates at 2 × 10^6^ cells per well. A mixture of gE, gD, and gC proteins (20 μg/mL) was added to the sample wells. A cell activation cocktail was added to the test wells, while complete RPMI 1640 medium was added to the blank wells. After a 2 h incubation at 37 °C with 5% CO_2_, brefeldin A (5 µg/mL) was added to the culture medium to block cytokine secretion, and the cells were incubated overnight. The next day, the cells were collected by centrifugation at 400× *g* for 5 min at 4 °C and then incubated with Zombie Aqua™ dye for 20 min at room temperature to stain viable cells. The cells were washed with staining buffer. After adding an anti-CD16/CD32 antibody (5 µg/mL) to block Fc receptors, the cells were incubated for 10 min at 4 °C, followed by the addition of PerCP/Cyanine 5.5 anti-mouse CD4 and FITC anti-mouse CD8a antibodies for 30 min at 4 °C. After being washed, the cells were fixed with fixation buffer for 20 min at room temperature, washed twice with permeabilization buffer, and incubated with PE anti-mouse IFN-γ and PE-Cy7 anti-mouse IL-2 antibodies for 1 h. A Cyto-FLEX flow cytometer (Beckman, Indianapolis, IN, USA) and FlowJo software V10.6.2 (BD, Franklin Lakes, NJ, USA) were used to analyze the proportions of IFN-γ and IL-2 secreting CD4^+^ and CD8^+^ T cells.

### 2.8. Determination of Viral Loads by Quantitative Polymerase Chain Reaction (qPCR)

The viral loads in vaginal swabs (6 mice per group) were quantified on days 2, 4, 6, 8, and 10 post HSV-2 infection. Furthermore, on day 6 after HSV-2 challenge, vaginal tissues (inoculation site) and key nervous system tissues (spinal cord and dorsal root ganglia of the sacrum) were collected from 4 mice per group. On day 5 post HSV-1 challenge, brain, trigeminal ganglion, and spinal cord were collected from 4 mice per group. Viral DNA was extracted using a MiniBEST Viral RNA/DNA Extraction Kit (Takara, Kusatsu, Shiga, Japan). The primers and probes used are listed in Table 2. The reaction was carried out using a Premix Ex Taq (Probe qPCR, Takara) kit. To standardize the viral load, the mean for the uninfected control group was subtracted from all results, and these normalized values were used for statistical analysis.

### 2.9. Data Analysis

Statistical analyses were conducted with GraphPad Prism 9.5.1 (GraphPad Software, La Jolla, CA, USA). Significant differences among groups were evaluated via one-way ANOVA, after which Tukey’s multiple comparisons test was employed to determine the significance of differences among the means of the different groups. Significance is indicated as follows: *p* > 0.05 (ns), *p* ≤ 0.05 (*), *p* ≤ 0.01 (**), *p* ≤ 0.001 (***), and *p* ≤ 0.0001 (****).

## 3. Results

### 3.1. Efficient mRNA Encapsulation by LNPs with Uniform Particle Size

**Figure 1:**
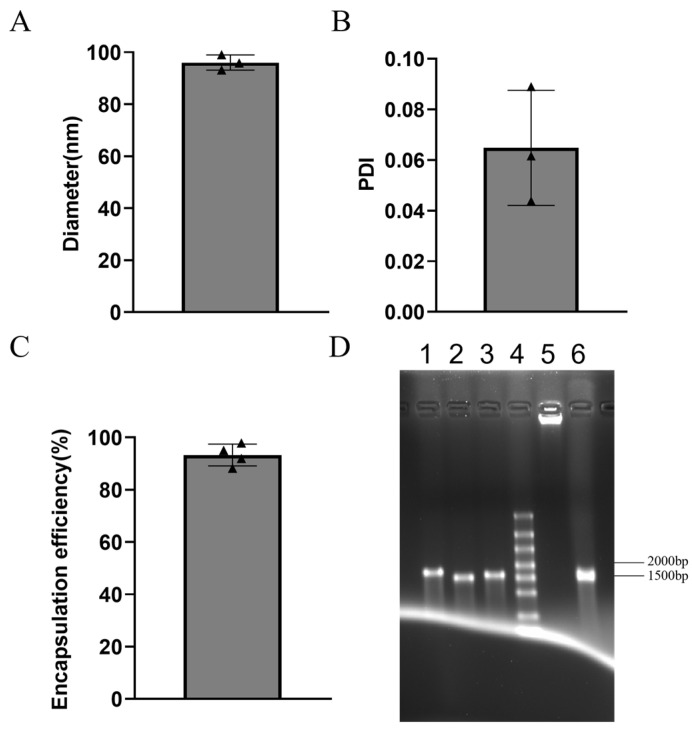
Characterization of three-component HSV-2 gE+gD+gC mRNA-LNP vaccines. (**A**) Particle diameter. (**B**) PDI. (**C**) Encapsulation efficiency. (**D**) Integrity assessment via formaldehyde-denaturing agarose gel electrophoresis. Lane 1: HSV-2 gE mRNA before encapsulation; lane 2: HSV-2 gD mRNA before encapsulation; lane 3: HSV-2 gC mRNA before encapsulation; lane 4: RNA marker; lane 5: mRNA-LNP complexes after encapsulation; lane 6: mRNA-LNP complexes after lysis.

After the mRNA aqueous phase and LNP lipid phase were encapsulated via a microfluidic device, the particle size, PDI, and integrity of the mRNA-LNP complexes were determined, and the mRNA encapsulation efficiency was calculated. The results from three consecutive tests revealed an average particle diameter of 96.03 nm (Figure 1A), which falls within an optimal range. The particle size significantly influences the biodistribution, cellular uptake, and endosomal escape of mRNA-LNPs; smaller LNPs typically exhibit increased tissue permeability and cellular uptake. The average PDI was measured at 0.06 (Figure 1B), indicating a highly uniform LNP particle size. The encapsulation efficiency was determined to be 93.25% (Figure 1C), demonstrating effective mRNA packaging. A high encapsulation efficiency is crucial for maintaining mRNA stability and payload integrity during formulation. Formaldehyde-denatured agarose gel electrophoresis was performed (Figure 1D), the premixed mRNA bands (lanes 1–3) appeared as single bands, indicating good integrity. The lysed mRNA-LNP complexes (lane 6) showed no smearing, and direct electrophoresis of the complexes (lane 5) resulted in no bands, confirming the absence of mRNA leakage and ensuring good integrity. In summary, these results indicate successful mRNA encapsulation in LNPs, making these LNPs suitable for subsequent animal experiments.

### 3.2. mRNA Vaccine and QS21 + CpG-Adjuvanted Subunit Vaccine Induced Superior Humoral Immune Response Compared to Aluminum-Adjuvanted Subunit Vaccine

**Figure 2:**
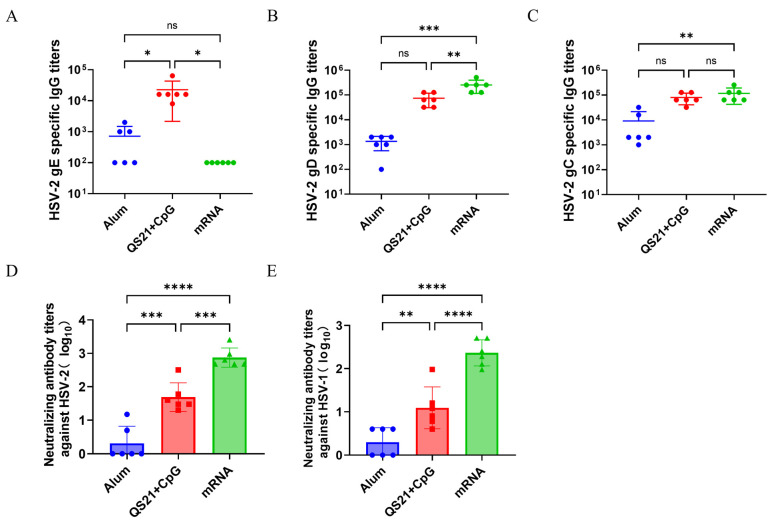
Measurement of antigen-specific antibody titers in serum by ELISA and neutralizing antibody titers by neutralization assay on day 7 after secondary immunization. (**A**) HSV-2 gE-specific IgG titer. (**B**) HSV-2 gD-specific IgG titer. (**C**) HSV-2 gC-specific IgG titer. (**D**) Neutralization titers against HSV-2. (**E**) Neutralization titers against HSV-1. ns: not significant; *: *p* ≤ 0.05; **: *p* ≤ 0.01; ***: *p* ≤ 0.001; ****: *p* ≤ 0.0001.

Titers of HSV-2 glycoprotein-specific IgG in mouse serum were quantified by ELISA on day 7 after secondary immunization. The QS21 + CpG-adjuvanted subunit vaccine resulted in the highest gE-specific IgG titer (22,666.7), which was significantly greater than that produced in response to the aluminum-adjuvanted vaccine (716.7, *p* ≤ 0.0001) (Figure 2A). Notably, the mRNA vaccine did not effectively induce the production of gE-specific antibodies. In contrast, the mRNA vaccine demonstrated the strongest ability to induce the production of gD-specific antibodies, resulting in the highest titer (256,000), followed by the QS21 + CpG-adjuvanted vaccine (74,666.7), whereas the aluminum-adjuvanted vaccine resulted in the lowest titer (1350) (Figure 2B). Similarly, the mRNA vaccine exerted the strongest effect in inducing the generation of gC-specific antibodies, resulting in the highest titer (117,333), followed by the QS21 + CpG-adjuvanted vaccine (80,000). The aluminum-adjuvanted vaccine resulted in the lowest titer (9166.7) (Figure 2C). Further, neutralizing antibody titers were determined to evaluate the capacity of antibodies in blocking virus infection. The mRNA vaccine induced the highest neutralizing antibody against HSV-2, with a geometric mean titer (GMT) of 747.14, followed by QS21 + CpG-adjuvanted vaccine (48.99) and both significantly higher than aluminum-adjuvanted vaccines (3.78) (Figure 2D). Surprisingly, the mRNA and QS21 + CpG-adjuvanted vaccines also induced very high neutralizing antibody titers against HSV-1, with a GMT of 232.59 and 12.34, while the aluminum-adjuvanted vaccine induced sera barely show cross-reactivity to HSV-1 with a GMT of 2.83 (Figure 2E). Overall, the mRNA vaccine and QS21 + CpG-adjuvanted vaccine induced robust humoral immune response in mice, with high neutralizing activity against both HSV-2 and HSV-1.

### 3.3. mRNA Vaccine and QS21 + CpG-Adjuvanted Vaccine Induced Robust Th1 Response

**Figure 3:**
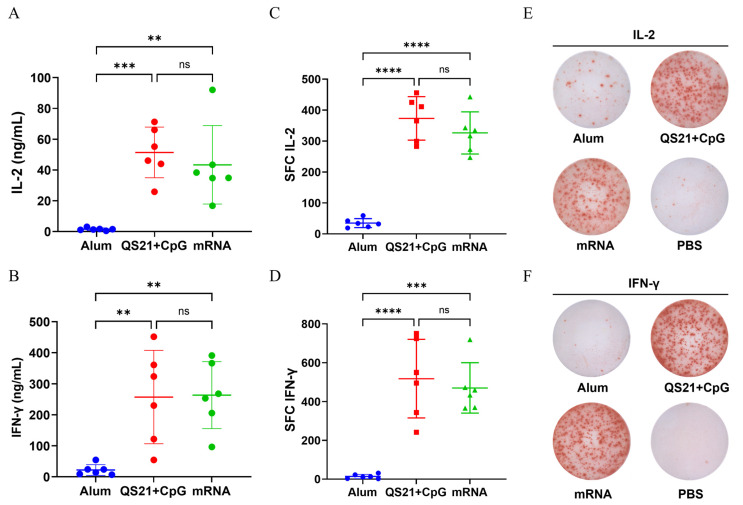
The cellular immune response levels in mice were measured by ELISA and ELISpot. (**A**) IL-2 concentration in splenocyte supernatant. (**B**) IFN-γ concentration in splenocyte supernatant. (**C**) Number of IL-2-secreting spot-forming cells (SFC). (**D**) Number of IFN-γ-secreting SFC. (**E**) Representative images of IL-2-secreting splenocytes. (**F**) Representative images of IFN-γ-secreting splenocytes. ns: not significant; **: *p* ≤ 0.01; ***: *p* ≤ 0.001; ****: *p* ≤ 0.0001.

Splenocytes were stimulated with HSV-2 gD, gE, and gC, then the level of IL-2 and IFN-γ in the supernatant were measured by ELISA to assess the strength of Th1 immune response. The QS21 + CpG-adjuvanted vaccine resulted in the highest IL-2 level (45.66 ng/mL), although the level of IL-2 in the mRNA vaccine (43.36 ng/mL) was comparable. There was no significant difference between the QS21 + CpG-adjuvanted vaccine and mRNA vaccine, but the IL-2 level in both these groups was significantly different from that in the aluminum-adjuvanted vaccine (1.51 ng/mL) (Figure 3A). Similarly, the QS21 + CpG-adjuvanted and mRNA vaccines induced comparable levels of IFN-γ secretion (257.08 ng/mL and 263.43 ng/mL, respectively), whereas the aluminum-adjuvanted vaccine resulted in the lowest level (22 ng/mL) (Figure 3B). To directly determine the number of IL-2- and IFN-γ-producing cells, we employed the ELISPOT assay. The results demonstrated that the QS21 + CpG-adjuvanted vaccine induced the highest number of antigen-specific IL-2-secreting splenocytes (mean spot number, 373.17), and the number of these splenocytes following immunization with the mRNA vaccine (326.33 spots) was comparable. Statistical analysis revealed no significant difference between these two vaccines but revealed significant differences between them and the aluminum-adjuvanted vaccine (34.83 spots) (Figure 3C). Similarly, the QS21 + CpG-adjuvanted vaccine (518 spots) and mRNA vaccine (470.33 spots) induced high levels of antigen-specific IFN-γ-secreting splenocytes, significantly higher than the aluminum-adjuvanted vaccine (13.67 spots) (Figure 3D).

**Figure 4:**
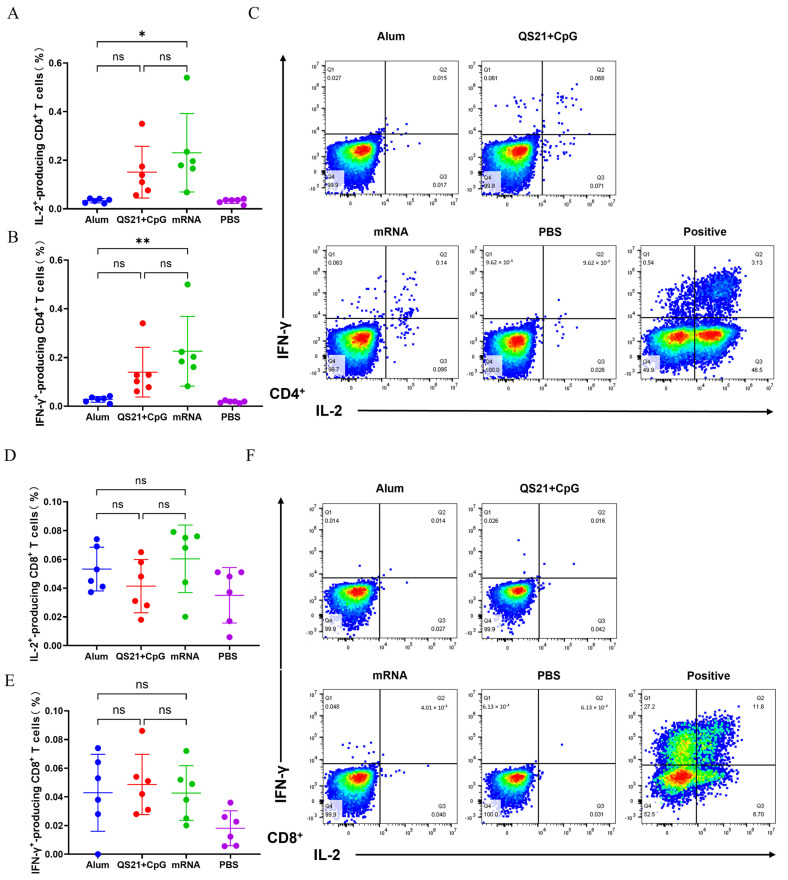
IL-2- and IFN-γ-producing CD4^+^ T cells and CD8^+^ T cells in splenocytes determined by flow cytometry. (**A**) Proportion of CD4^+^ T cells secreting IL-2. (**B**) Proportion of CD4^+^ T cells secreting IFN-γ. (**C**) Representative plots of CD4^+^ T cells secreting IL-2 and IFN-γ. (**D**) Proportion of CD8^+^ T cells secreting IL-2. (**E**) Proportion of CD8^+^ T cells secreting IFN-γ. (**F**) Representative plots of CD8^+^ T cells secreting IL-2 and IFN-γ. ns: not significant; *: *p* ≤ 0.05; **: *p* ≤ 0.01.

We further used flow cytometry to investigate the subset of IL-2- and IFN-γ-producing T cells. The results showed that the mRNA vaccine induced IL-2- and IFN-γ-secreting CD4^+^ T cells at a frequency of 0.230% and 0.225%, while the QS21 + CpG-adjuvanted vaccine induced these cells at 0.150% and 0.139%. In contrast, the aluminum-adjuvanted vaccine induced a frequency lower, to 0.033% and 0.028% (Figure 4A,B). All three forms of vaccine induced very weak antigen-specific IL-2- and IFN-γ-secreting CD8^+^ T cells (Figure 4D,E), demonstrating these HSV-2 vaccine primarily exert CMI via Th1 cell induction, with limited effects on cytotoxic CD8^+^ T cell activation. Collectively, these findings demonstrated that the QS21 + CpG-adjuvant and mRNA vaccines induced robust Th1 cellular immune response, whereas the aluminum-adjuvanted subunit vaccine fails to elicit significant Th1-type cytokine secretion.

### 3.4. QS21 + CpG-Adjuvanted Subunit Vaccine and mRNA Vaccine Protected Mice from Lethal HSV-2 Challenge

**Figure 5:**
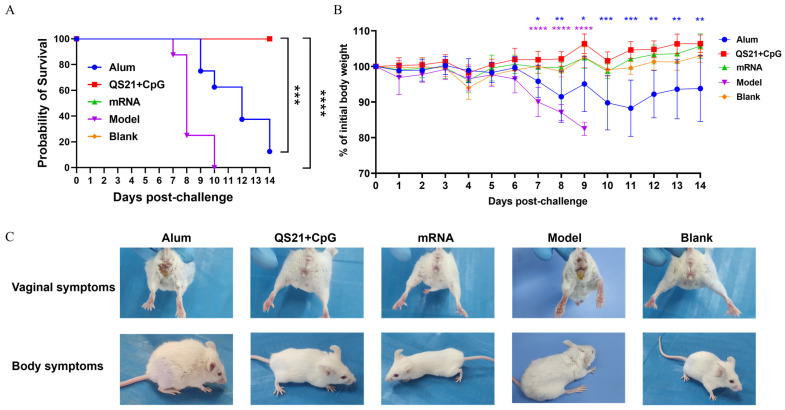
Clinical manifestations of the mice in each vaccine group after HSV-2 challenge. (**A**) Survival rate of mice post-challenge. (**B**) Weight changes of mice post-challenge. (**C**) Representative images of the clinical symptoms of mice post-challenge. ns: not significant; *: *p* ≤ 0.05; **: *p* ≤ 0.01; ***: *p* ≤ 0.001; ****: *p* ≤ 0.0001.

To further evaluate the immune response induced by these vaccines, we challenged immunized mice or unimmunized mice (model group) with a lethal dose of HSV-2. As shown in Figure 5A, the survival rate of the model group began to decline from day 7 post-challenge, with all the mice succumbed by day 10. In vaccinated animals, the aluminum-adjuvanted vaccine group had a survival rate of 12.5% by day 14, whereas both the QS21 + CpG-adjuvanted and the mRNA vaccine groups exhibited 100% survival, indicating the effectiveness of these vaccines in protecting mice from lethal viral challenge. Compared with the uninfected blank group, the model group presented significant weight loss from day 7 (*p* ≤ 0.0001) until all the mice died (day 10). The aluminum-adjuvanted group presented significant weight loss beginning on day 7 (*p* ≤ 0.05); moreover, the decrease in weight worsened over time, and the weight of the aluminum-adjuvanted vaccine group was lower than that of the blank group by day 14 (*p* ≤ 0.01). In stark contrast, the QS21 + CpG-adjuvanted and mRNA vaccine groups presented no significant weight loss, indicating that the lethal dose of the virus had no impact (Figure 5B). Meanwhile, the model group rapidly developed severe symptoms, including vaginal swelling, fur roughness, constipation, and hind-limb paralysis. The aluminum-adjuvanted vaccine group exhibited similar symptoms. However, the QS21 + CpG-adjuvanted and mRNA vaccine groups presented no clinical symptoms at all (Figure 5C). In summary, these results indicate that, in mice, both the three-antigen QS21 + CpG-adjuvanted vaccine and mRNA vaccine effectively protected against lethal viral challenge, achieving complete protection. In contrast, the aluminum-adjuvanted vaccine did not provide adequate protection.

### 3.5. Viral Load Assessment Demonstrated Robust In Vivo Protection of QS21 + CpG-Adjuvanted and mRNA Vaccines

**Figure 6:**
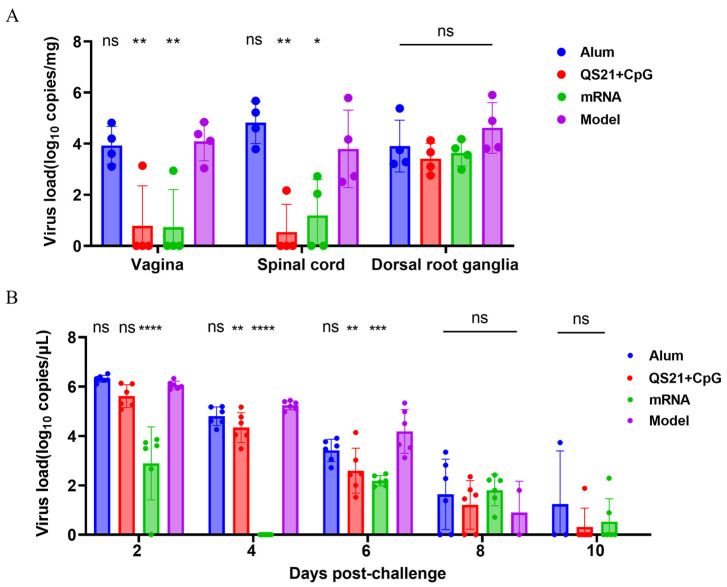
Determination of viral load in tissues by real-time qPCR after HSV-2 challenge. (**A**) Viral loads in tissues on day 6 post-challenge. (**B**) Viral loads in vaginal swab at indicated time points. Statistical significance was calculated using one-way ANOVA, followed by Dunnett’s multiple comparisons test, with the model group serving as the control. ns: not significant; *: *p* ≤ 0.05; **: *p* ≤ 0.01; ***: *p* ≤ 0.001; ****: *p* ≤ 0.0001.

On day 6 after HSV-2 challenge, the viral load in mouse vaginal, spinal cord, and dorsal root ganglion tissues were measured (Figure 6A). Real-time qPCR results showed that, compared with those in the model group, the viral load in vaginal tissues in the QS21 + CpG-adjuvanted and mRNA vaccine groups was 77.38-fold and 121.14-fold lower, respectively, while that in the aluminum-adjuvanted group was only 1.22-fold lower. The viral load in spinal cord tissues was 4288.82-fold and 982.02-fold lower in the QS21 + CpG-adjuvanted and mRNA groups than in the model group but was greater in the aluminum-adjuvant group than in the model group. The viral load in the dorsal root ganglia was 45.16-fold, 34.11-fold, and 3.61-fold lower in the QS21 + CpG-adjuvanted, mRNA, and aluminum groups, respectively, than in the model group. To evaluate the shedding of the virus at primary infection sites, vaginal swabs were taken on days 2, 4, 6, 8, and 10 post-challenge to measure the viral loads (Figure 6B). On day 2, the mRNA vaccine group presented a significantly lower viral load than the model group did (*p* ≤ 0.0001), with a 362.45-fold reduction. Compared with the model group, the QS21 + CpG-adjuvanted group presented a 1.94-fold reduction, whereas the viral load in the aluminum group was even greater than that in the model group. By day 4, no virus was detected in the mRNA vaccine group, indicating nearly complete restriction on virus shedding. The QS21 + CpG-adjuvanted group also presented significantly lower viral load than the model group did, with reductions of 4.24-fold (*p* ≤ 0.01), while the aluminum-adjuvanted group reduced it by only 2.25-fold (ns). On day 6, the mRNA vaccine, QS21 + CpG-adjuvanted, and aluminum-adjuvanted groups presented viral load reductions of 350.44-fold (*p* ≤ 0.001), 22.76-fold (*p* ≤ 0.01), and 15.82-fold (ns), respectively. From days 8 to 10, the viral loads in all the groups were very low, with no significant differences among the groups.

Taken together, these results indicated that immune response elicited by the QS21 + CpG-adjuvanted and mRNA vaccines can inhibit the replication of the invading HSV-2 virus in vivo, with the mRNA vaccine showing the best efficacy in preventing virus shedding at primary infection sites. In contrast, the aluminum-adjuvanted vaccine only showed marginal inhibitory effects in restricting viral replication.

### 3.6. The QS21 + CpG-Adjuvanted Subunit Vaccine and mRNA Vaccine Provided Cross-Protection Against HSV-1 Infection

**Figure 7:**
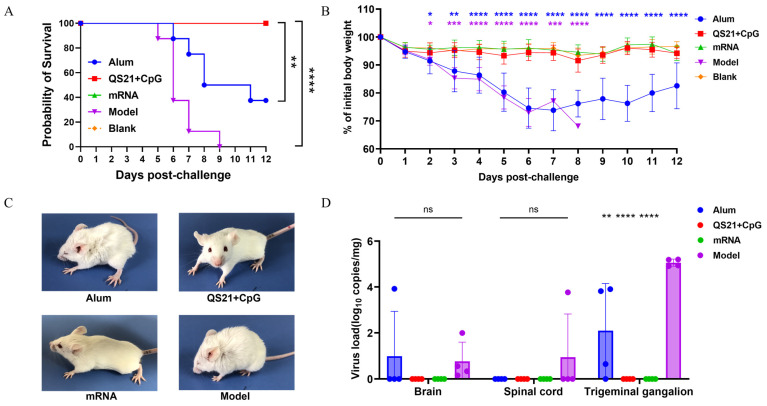
Clinical manifestations and viral load in tissues after HSV-1 challenge. (**A**) Survival rate of mice post-challenge. (**B**) Weight changes in the mice post-challenge. (**C**) Representative images of clinical symptoms in mice post-challenge. (**D**) Viral load in tissues measured by real-time qPCR. Viral load data were compared using one-way ANOVA, followed by Dunnett’s multiple comparisons test, with the model group serving as the control. ns: not significant; *: *p* ≤ 0.05; **: *p* ≤ 0.01; ***: *p* ≤ 0.001; ****: *p* ≤ 0.0001.

HSV-1 is a closely related pathogen to HSV-2, though showing different tissue tropism. To evaluate if the trivalent HSV-2 vaccines provide cross-protective effect to HSV-1 infection, we challenged mice that immunized with HSV-2 vaccines with a lethal dose of HSV-1. Five days after challenge, the model group began to die, and all succumbed by day 9. The aluminum-adjuvanted vaccine group reached a mortality of 62.5% by day 12; however, both the QS21 + CpG-adjuvanted and mRNA vaccine groups maintained 100% survival (Figure 7A). The aluminum-adjuvanted group, with similarity to the model group, showed significant weight loss from day 2 (*p* ≤ 0.05) that continued to worsen, remaining significantly lower than the blank group by day 12 (*p* ≤ 0.0001). In contrast, the QS21 + CpG-adjuvanted and mRNA vaccine groups demonstrated no significant weight loss (Figure 7B). Consistent with the survival rate and weight change, the model group developed symptoms such as fur roughness, arched back, keratitis, reduced mobility, and even paralysis. Similarly, the mice in aluminum-adjuvanted vaccine group all displayed the same acute clinical symptoms. In contrast, the mice in the QS21 + CpG-adjuvanted and mRNA vaccine groups exhibited none of these symptoms (Figure 7C). By day 5 after challenge, the HSV-1 virus had substantial replication in the brain, spinal cord, and especially the trigeminal ganglion of mice in the model group; however, the QS21 + CpG-adjuvanted and mRNA vaccine-immunized animals showed no trace of virus in these tissues. In contrast, the aluminum-adjuvanted vaccine group only showed marginal suppression of HSV-1 replication in brain and TG (Figure 7D). These findings are consistent with the symptom manifestations and indicated that the HSV-2 QS21 + CpG-adjuvanted subunit and mRNA vaccines can induce effective cross-protection against HSV-1 in immunized mice, highlighting the excellent protective efficacy of these trivalent HSV-2 vaccines.

## 4. Discussion

Given HSV-2 gD’s role in host cell entry, gE’s function in cell-to-cell spread and immune evasion, and gC’s capacity of mediating complement evasion, a gE + gD + gC trivalent vaccine strategy was employed in this study, using aluminum hydroxide or QS21 + CpG as adjuvants, or in the form of a gE + gD + gC mRNA vaccine. Results from humoral immune response revealed that the QS21 + CpG-adjuvanted subunit vaccine successfully induced high levels of gE-, gD-, and gC-specific antibodies. By contrast, the aluminum-adjuvanted vaccine induced lower levels of antibodies, and the mRNA vaccine induced high levels of gD- and gC-specific antibodies but failed to induce detectable gE-specific antibodies. This discrepancy may have resulted from various factors, including mRNA delivery efficiency, stability, translation efficiency, antigen immunogenicity, and immune response dynamics. In terms of serum neutralization, the mRNA vaccine was found to result in the highest GMT against HSV-2, and the QS21 + CpG-adjuvanted vaccine also exhibited excellent neutralizing antibody induction. Meanwhile, the aluminum-adjuvanted vaccine elicited marginal levels of neutralizing activity. Since gD is the major neutralization target, the greater in vitro neutralizing ability of the mRNA vaccine compared to the subunit vaccine is consistent with their capacity to induce gD-specific IgG.

Apart from inducing strong humoral immune response, the QS21 + CpG-adjuvanted vaccine and mRNA vaccine also induced robust antigen-specific CMI, and similar to the VZV vaccine [28,29,30], the CMI of these two vaccines was primarily mediated by Th1-type CD4^+^ T cell responses, whereas the aluminum-adjuvanted vaccine failed to induce effective CMI.

In agreement with the potent humoral and cellular immune responses elicited by the QS21 + CpG-adjuvanted subunit vaccine and mRNA vaccine, both the vaccines protected mice from lethal HSV-2 challenge, with a high efficiency of restricting virus replication in neural tissues and viral shedding in vaginal tissue. Corresponding to its immunogenicity, the aluminum-adjuvanted vaccine did not protect mice from lethal viral challenge, showing no difference in tissue viral loads from the model group. Notably, during the initial stages of infection, the mRNA vaccine demonstrated the fastest virus-clearing capability at the infection site, likely related to the high level of neutralizing antibody it induced.

In this study, both the QS21 + CpG-adjuvanted vaccine and mRNA vaccine provided excellent cross-protection against HSV-1 infection, while the aluminum-adjuvanted vaccine failed to offer effective protection. This further confirms that these two vaccines can induce broad-spectrum neutralizing antibodies targeting conserved antigenic epitopes on HSV-1 and HSV-2. Moreover, these vaccines activated adaptive T-cell responses, producing high levels of IL-2 and IFN-γ, which are crucial for the clearance of invading viruses. Inducing cross-protection is meaningful for the development of the HSV vaccine. Given that HSV-1 and HSV-2 share about 74% nucleotide homology and similar glycoprotein composition [31], they can infect beyond their typical regions [32]. HSV-2 can cause oral herpes, while HSV-1 may trigger genital herpes [33,34]. Therefore, a vaccine that provides protection against both viruses would enhance its practicality and public health value.

Recent advances in mRNA vaccine technology have provided valuable insights and context for our study. For instance, a 2023 study demonstrated that a trivalent mRNA vaccine based on HSV-2 is effective against both HSV-2 and offers robust protection against HSV-1 infection [35]. Additionally, another study highlighted the immune mechanisms activated by an mRNA vaccine encoding gE, gD, and gC, which is currently in a phase I clinical trial [36] (ClinicalTrials.gov NCT05432583), further supporting the rationale of our approach. Collectively, these studies underscore the novelty and relevance of our work in utilizing an mRNA vaccine platform for HSV-2, providing a broader framework for understanding the potential of mRNA vaccines in combating viral infections, including HSV-2 and HSV-1. While the protein-based vaccine benefits from well-established manufacturing processes and good storage stability, advantageous for large-scale distribution and administration, the mRNA vaccine platform presents innovative advantages. The mRNA vaccine enables rapid development, can encode multiple antigens within a single formulation, and does not require adjuvants that may raise safety concerns. However, it also presents challenges, such as the need for cold chain storage and the technical complexity of ensuring efficient in vivo delivery and translation of the mRNA. The choice between these two platforms should be based on specific research objectives, target populations, and practical factors such as manufacturing capabilities and distribution logistics. Our study compares these two vaccine platforms, emphasizing their respective strengths and limitations in the development of an HSV-2 vaccine.

In summary, the gE, gD, gC trivalent HSV-2 vaccine, in the forms of QS21 + CpG-adjuvanted subunit vaccine or mRNA vaccine, showed promising immunogenicity and efficient protective activity in mice, offering significant insights for future HSV-2 vaccine development. However, challenges remain in precisely selecting and combining antigens to balance humoral and cellular immune responses while avoiding potential side effects. Additionally, the acute infection mouse model used here is mainly utilized for evaluating the efficacy of preventive vaccines. Given the high prevalence of HSV-2 infection, therapeutic vaccines are urgently needed. In future work, we will evaluate these vaccines in latently infected guinea pig models as therapeutic vaccines, with the aim of meeting broader clinical needs.

## 5. Conclusions

This study comprehensively assessed the immunogenicity and protective effects of trivalent HSV-2 vaccines with the gE, gD, and gC antigens under different adjuvants and vaccine forms. The results showed that the QS21 + CpG-adjuvanted subunit vaccine and mRNA vaccine effectively induced humoral and cellular immune responses, significantly reducing viral loads in HSV-2- and HSV-1-infected mice. In contrast, the aluminum-adjuvanted vaccine performed poorly, indicating the limitations of traditional aluminum adjuvants in activating protective immunity. This research not only confirmed the effectiveness of combining gE, gD, and gC triple antigens but also demonstrated the potential of QS21 + CpG-adjuvanted and mRNA vaccines in the development of HSV-2 vaccine.

## Figures and Tables

**Table 1:** Vaccine groups and dose per injection.

Vaccine Group	gE + gD + gC	Alum	QS21	CpG 1018S	mRNA
1.Alum	7.5 μg	50 μg	-	-	-
2.QS21 + CpG	7.5 μg	-	5 μg	10 μg	-
3.mRNA	-	-	-	-	15 μg
4.PBS	-	-	-	-	-
5.Model	-	-	-	-	-

- Not added.

**Table 2:** Primers and probes.

Name	Sequence
HSV-2-gG-F	CGCTCTCGTAAATGCTTCCCT
HSV-2-gG-R	TCTACCCACAACAGACCCACG
HSV-2-gG-Probe	6-FAM-CGCGGAGACATTCGAGTACCAGATCG-BHQ1
HSV-1-UL7-F	GGTGCCGGTTGCGGTTCGT
HSV-1-UL7-R	GAACCGGTGCAGCAGAACG
HSV-1-UL7-Probe	FAM-AGTCCCGAGGACGCCTATGTGACG-TAMRA

## Data Availability

All data used during the study are available from the corresponding author by request.
